# Nanopore sequencing: An enrichment‐free alternative to mitochondrial DNA sequencing

**DOI:** 10.1002/elps.201800083

**Published:** 2018-12-13

**Authors:** Roxanne R. Zascavage, Kelcie Thorson, John V. Planz

**Affiliations:** ^1^ Department of Microbiology Immunology and Genetics University of North Texas Health Science Center Fort Worth TX USA; ^2^ Department of Criminology and Criminal Justice University of Texas at Arlington Arlington TX USA; ^3^ Zoetis Inc. Parsippany NJ USA

**Keywords:** MinION, mtDNA, Nanopore, Sequencing

## Abstract

Mitochondrial DNA sequence data are often utilized in disease studies, conservation genetics and forensic identification. The current approaches for sequencing the full mtGenome typically require several rounds of PCR enrichment during Sanger or MPS protocols followed by fairly tedious assembly and analysis. Here we describe an efficient approach to sequencing directly from genomic DNA samples without prior enrichment or extensive library preparation steps. A comparison is made between libraries sequenced directly from native DNA and the same samples sequenced from libraries generated with nine overlapping mtDNA amplicons on the Oxford Nanopore MinION™ device. The native and amplicon library preparation methods and alternative base calling strategies were assessed to establish error rates and identify trends of discordance between the two library preparation approaches. For the complete mtGenome, 16 569 nucleotides, an overall error rate of approximately 1.00% was observed. As expected with mtDNA, the majority of error was detected in homopolymeric regions. The use of a modified basecaller that corrects for ambiguous signal in homopolymeric stretches reduced the error rate for both library preparation methods to approximately 0.30%. Our study indicates that direct mtDNA sequencing from native DNA on the MinION™ device provides comparable results to those obtained from common mtDNA sequencing methods and is a reliable alternative to approaches using PCR‐enriched libraries.

**Abbreviations**
kbp: kilobase pairs
MPS: Massively Parallel Sequencing
mtDNA: mitochondrial DNA
mtGenome: mitochondrial genome
ssDNA: single strand of DNA

## 1 Introduction

The mitochondrial genome has been of great interest across diverse disciplines of biological research since it was first identified in the 1960s 1, 2. In vertebrates, the circular genome varies in size from approximately 11 kbp – 28 kbp, however plant and invertebrate genomes can be considerably larger 3, 4. The content of typical animal mtGenomes consists of 37 genes coding for protein subunits for the Oxidative Phosphorylation pathway, ribosomal RNAs and mitochondria‐specific transfer RNAs (tRNA) in addition to approximately 1–2 kbp of non‐coding regulatory sequences 5. The evaluation of mtGenomes has provided considerable insight into the evolution of organisms and of genomes at the inter‐ and intraspecific level 6, 7, 8. Due to the ease of isolation of the mitochondria and simplification of mtDNA extraction techniques, early studies utilizing restriction endonuclease digestion and gel electrophoresis began to explore sequence diversity within and between populations and closely associated species, including humans 7, 9, 10, 11. Development of robust DNA sequencing approaches 12, 13 fostered the completion of the human mtGenome build in 1982 14. Subsequent corrections to the reference sequence by Andrews et al. 15 ushered in concerted efforts to characterize the mtGenomes of numerous other organisms 5, anthropological samples, and species of special concern. Availability of mtGenomes supported the selection of a 648 base‐pair region of the mitochondrial cytochrome c oxidase 1 gene (CO1) as the standard barcode region for the International Barcode of Life (iBOL) database [16, http://www.ibol.org/] which is capable of distinguishing the majority of animal species 17, 18.

The introduction of PCR‐based sequencing 19 and high‐throughput capillary electrophoresis methods 20 increased the practicality of examining specific regions of the mtGenome and nuclear genome across all biological and biomedical disciplines. The current Massively Parallel Sequencing (MPS) approaches have ever improved the sequencing processes, allowing high–order statistical sampling of individual sequence strands generated through PCR enrichment from targeted sites of interest throughout the mitochondrial and nuclear genomes with applications in gene discovery 21, 22, human identification 23, 24, 25, functional genomics 26, 27, and clinical genetics 28, 29. Sequencing robustness of the MPS processes relies on extensive read depths through the target region to statistically overcome the inherent errors of the sequencing chemistries 30, 31. PCR‐induced errors can occur when samples contain nucleotide modifications, such a methylation or oxidation 32, 33, or when DNA is damaged during the sample and library preparation processes 34.

In 1989, David Deamer proposed that a single strand of DNA (ssDNA) could be sequenced by using voltage to ‘pull’ the strand through a nanoscopic pore 35. A nanopore consists of a protein with a hollow tube that is approximately 1nm in diameter. The nanopore protein is inserted across a synthetic polymer membrane. The membrane has high electrical resistance and an ionic current is generated through the nanopore protein by applying an electrical potential across the membrane. Molecules entering the nanopore cause changes in the ionic current. The disruptions can be quantitatively measured to identify the molecule 36. Nanopore sequencing of DNA requires an enzyme complex that attaches to the nanopore on the *cis* side of the membrane with the DNA library strand. The enzyme acts as a regulator or “ratchet” to facilitate DNA strand entry into the nanopore and control the speed of the translocation. A ssDNA strand is fed through the nanopore migrating to the positively‐charged *trans* side of the membrane. The smallest area of the pore opening undergoes conformational changes as individual DNA molecules pass through, and fluctuations in the electrical field are detected. This aperture is responsible for base discrimination 35.

In 2012, Oxford Nanopore Technologies (ONT) introduced the MinION device utilizing nanopore‐based sequencing capable of sequencing DNA, RNA and proteins. The device was first released to a collaborative worldwide MinION Access Program (MAP) in 2014. Since then, the MinION device, nanopore‐housing flowcells, and the operational and basecaller softwares have gone through multiple iterations, each improving its read accuracy and performance 37, 38, 39. Laboratories worldwide have adopted the MinION system as an alternative sequencing strategy to conventional methods and have developed workflows ranging from microbial detection in environmental samples, to *de novo* genome sequencing of non‐model species, and human gene methylation studies from clinical samples 30, 31, 32, 33, 34, 35, 36, 37, 38, 39, 40, 41, 42, 43, 44, 45, 46. The platform's streamlined library workflows, lower costs, and portability provide advantages for some applications over conventional MPS approaches.

A variety of library preparation methods have been introduced for the MinION platform. A standard two‐adaptor (1D) chemistry permits adaptor ligation to DNA fragments generated through mechanical shearing or PCR amplification. Each strand of the fragment is sequenced independently as strands are recruited into the nanopores. A hairpin‐containing 2D library approach permits continuous strand sequencing of both the template strand and its complement through their linkage via a hairpin adaptor. Both approaches accommodate barcoded linkers to allow for multiplexing individual samples. A 1D^2^ library kit that promotes the association of the two individual strands such that a high percentage of the template strand and its complement are sequenced sequentially, as well as a transposase‐mediated Rapid 1D library system were also recently introduced.

A major benefit of nanopore sequencing is that libraries are not restricted based on the capabilities of the sequencing chemistry/instrumentation as in Illumina or ION Torrent systems. Successful sequencing of DNA strands 2–40 kbp in length are routinely obtained using the 1D‐style chemistries from high quality DNA preparations or amplicon libraries. The transposon‐based Rapid 1D chemistry produces genomic DNA libraries composed of randomly sized fragments, with fragments larger than 882 kbp observed 36. The longer read lengths possible with the MinION platform permit phasing of SNPs in regions that are captured as single reads spanning multiple kilobases, as well as serving as an architectural scaffold for more accurate assemble of novel genomes or genes sequenced utilizing the short read MPS processes 47. The MinION device has been used to characterize microbiomes using 16S rRNA sequences 48 and Zika, Ebola and other viral genomes are being directly sequenced from clinical samples 42, 43. Recently, Chandler et al. 48 reported *de novo* sequencing and assembly of the mitochondrial genome of a nematode parasite used as a model organism in human hookworm infection. Using an approach similar to Chandler et al. 48, Lindberg et al. 49 reported on the utility of the MinION device in distinguishing mixtures in the human mitochondrial genome used in conjunction with Illumina MiSeq data. As identified in the study, the short read length capacity of the MiSeq data prevented proper phasing of SNP polymorphisms in the mtGenome to allow for effective mixture deconvolution. The deconvolution of mtDNA heteroplasmy is of significant interest in numerous mitochondria‐implicated diseases 50, 51, 52, ancient DNA studies 53, and forensic investigations 53, 54, 55, 56.

The goals of this study were to determine the efficacy of sequencing full Human mitochondrial genomes utilizing the ONT MinION™ device and evaluate the accuracy of this DNA sequencing platform by direct comparison to the sequence of a NIST‐traceable mtDNA sequencing standard. Sequencing results obtained from native (non‐enriched) genomic DNA samples and sample libraries in which the mtGenomes were enriched through PCR are directly compared to evaluate the efficacy of both methods for clinical or forensic samples. Comparisons will be drawn from data generated with various versions of the MinION flowcells, sequencing library chemistries and collection/basecalling software versions implemented during this project.

## 2 Materials and methods

### 2.1 Sample preparation

Genomic DNA (gDNA) was extracted from eight individual liquid blood samples using the QIAamp® Mini Blood Kit (QIAGEN, Hilden, Germany) according to manufacturer's specifications. All samples were eluted to a final volume of 200 μL. The quality and quantity of each extracted DNA sample was assessed on the Agilent^©^ 4200 Bioanalyzer (Agilent Technologies, Santa Clara, CA, USA) using the Genomic DNA kit according to the manufacturer's specifications. If needed, recovered DNA was concentrated using Microcon®100, eluted to 40 μL, and then quantified. Extracted DNA from human cell line HL‐60, obtained from ATCC® (Manassas, VA, CCL‐240D™), served as a standard NIST‐traceable control DNA. Collection of blood samples used in this study was approved by the Institutional Review Board of the University of North Texas Health Science Center (Protocol #2010‐106).

### 2.2 Preparation of PCR amplicon sequencing libraries

Overlapping PCR amplicons covering the entire mitochondrial genome were generated using an adaptation of single‐plex primers reported by Ramos et al. 57. PCR was performed with the TaKaRa LA PCR Kit (TaKaRa Bio, Otsu, Shiga, Japan) following the manufacturer's specifications, with an input of 0.4ng of DNA. PCR was performed on a Mastercyler® Pro S (Eppendorf, Hamburg, Germany) using the following thermal‐cycling parameters: an initial temperature of 94°C for 1 min; followed by 36 cycles of 94°C for 30 s and 54°C for 2 min 15s. After cycling, there was a final extension step of 72°C for 10 min. Amplicons were assessed and quantified on the Agilent^©^ 4200 Bioanalyzer using the D5000 kit according to the manufacturer's specifications. The amplicons were normalized to 5 nmol/25μL in nuclease‐free water.

The nine PCR amplicon pools for each sample were combined for a total volume of 225 μL. Each sample was then purified with QIAquick® PCR Purification Kit (QIAGEN, Hilden, Germany) and eluted to 50 μL. The total amount of amplified DNA in combined amplicon sets was determined using the Agilent^©^ 4200 TapeStation using the D5000 kit. The amplicon pools for the eight samples were individually barcoded using the ONT EXP‐NDB002 barcoding kit following manufacturer's specifications. The barcoded samples were pooled and prepared for sequencing using the Oxford Nanopore Technology SQK‐NSK007 sequencing kit v9 (2D sequencing). HL‐60 was prepared as a single library and not barcoded or combined with the other samples.

### 2.3 Preparation of whole genome sequencing libraries

Sequencing libraries from native genomic DNA without enrichment were generated using SQK‐RAD001 kit v9 or SQK‐RAD002 with EXP‐LLB001 (1D sequencing), each according to the manufacturer's protocol with the following modifications: The manufacturer recommendation is 200 ng of input DNA. Input DNA amount varied from 144.8 to 259.5 ng.; In order to improve ligation efficiency, the amount of Blunt/TA ligase added was increased from 0.2 to 0.4 μL with an extended room temperature incubation of 10 min.

### 2.4 DNA library sequencing

#### 2.4.1 Amplicon library sequencing

Amplified libraries were sequenced on MKI vR9 MinION flow cells. Flow cells were primed with a mixture of 500 μL nuclease‐free water and 500 μL RBF1 prior to library loading. The library was applied directly into the sample port on flow cell using a 1000 μL pipette. Flow cells were placed in the MinION™ device and sequenced for a total of 48 h, with reloading done at 24 h, using ONT MinKNOW software v1.4.2.

#### 2.4.2 Whole genome DNA library sequencing

Native DNA libraries were sequenced on MKI vR9.4 SpotON MinION flow cells. Flow cells were primed with a mixture of 520 μL nuclease‐free water and 480 μL RBF1 prior to library loading. Libraries were loaded in a drop‐wise fashion into the SpotON port of the flow cell. Flow cells were placed in the MinION™ device and sequenced for a total of 48 h using ONT MinKNOW software v1.4.2.

### 2.5 Sequencing data basecalling

Basecalling was performed by one of three interchangeable base calling software programs provided through ONT: C++ program Albacore v0.9.1, cloud‐based Metrichor v2.45.3, or ONT's real‐time local base caller. The variation in basecaller selection was due to the progression of basecaller platforms from a cloud‐based method, to a local method, to a real‐time local method over the course of this study. All three programs utilize a comparable base calling algorithm and output the results into a standard FAST5 format. Several datasets were basecalled with all three platforms in order to ensure concordance (data not provided, but available upon request). With the exception of the improved homopolymeric correction outlined in this study, basecaller selection had no impact on the consensus sequence results.

### 2.6 Sequencing read alignment, pile up, and consensus generation

Nanopolish v0.6.0 41 was used to extract the reads from the FAST5 data files and convert them to FASTA files. BWA‐MEM v0.7.12‐r1039 58 was used to align the sequencing data to the revised Cambridge reference sequence (rCRS) and then create a SAM file of the alignment. SAMtools v1.3.1 59 converted the SAM file to BAM file. Tablet v1.16.09.16 60 was used to visually inspect the pileup in alignment with the rCRS. BAM files were used with a SAMtools script, mpileup 59, and aligned to rCRS to generate a variant table of base calls for all positions of the mtGenome. The read with the highest number of calls at each position was identified and registered as the official base for that position in the consensus sequence. The NIST‐traceable control sample, HL‐60, was additionally aligned to the known mtDNA sequence for itself instead of the rCRS in order to evaluate sequencing accuracy.

### 2.7 Sequencing method comparison

Native and amplicon consensus sequences were aligned to each other via a map to reference alignment with rCRS as the reference genome in Geneious v.10.1.3 61. Total number of identical bases and differences between the consensus sequences were calculated. Types of differences were recorded. In order to evaluate error rate of each sequencing method, HL60 consensus sequences were mapped to its known reference sequence in Geneious. Number and type of errors detected relative to the reference were evaluated.

### 2.8 Homopolymeric correction assessment

There are several regions within the mtGenome that contain homopolymeric stretches that can create difficulties in PCR, synthesis‐based sequencing, or strand reading in nanopore sequencing 62. Within the human mtGenome there are cytosine (C), adenine (A) stretches that extend beyond 3 repeats that are prone to sequencing errors with the incorporation/detection of fewer or additional nucleotides than occur in the native DNA molecules. HL‐60 native and amplified sequencing results were re‐basecalled using Albacore, following the release of v1.1, which included a homopolymeric correcting algorithm. The newly basecalled data was evaluated according to the methods outlined in 2.6 and 2.7. Final assessments included an alignment of the pre‐homopolymeric correction to post‐homopolymeric correction to assess the value of the modified algorithm.

## 3 Results and discussion

### 3.1 Depth of coverage

Sequencing data was analyzed to assess depth of coverage necessary to obtain accurate consensus data. With the exception of HL60, which was used to generate an independent library, all of the enriched samples were pooled and barcoded. With six samples represented in a single library, the number of total reads per sample was less than 10 000. However, because the pool was made up of targeted mitochondrial amplicons, the majority of reads mapped to the reference genome, therefore generating a greater depth of coverage than that observed with the native samples. Average depth of coverage for barcoded, PCR enriched samples ranged from 295x‐813x. The lowest coverage for a single position in any amplified sample was 54x (Table 1).

**Table 1:** Depth of coverage of the mitochondrial genome was assessed for whole genome DNA libraries and PCR enriched DNA libraries

	Native DNA samples					PCR enriched DNA samples				
Sample	Input DNA (in ng)	Reads*	Mapped reads	Average coverage	Coverage range	Input DNA (in ng)	Reads*	Mapped reads	Average coverage	Coverage range
HL60	212	307 277	888	118	71–139	0.4	314 567	227 279	20 417	3213—47 838
101	144.8	297 624	429	92	47–113	0.4	6135	6105	686	52–2042
102	229.5	96 710	281	39	23–49	0.4	2557	2550	295	54–784
103	207	160 337	390	69	42–82	0.4	4357	4344	507	123–1274
433	176.3	222 357	490	33	15–44	0.4	3431	3423	399	74–1004
441	259.5	258 867	230	37	20–52	0.4	6178	6114	743	180–1555
442	237	235 930	280	70	42–82	0.4	6835	6767	813	201–1789
449	204	179 255	184	15	8–20	0.4	2874	2852	334	80–812
459	234	175 492	230	24	12–33	0.4	4393	4365	487	75–1414

^*^Reads reflect the number of reads to pass the quality filter of the basecaller for a particular sample. This is not the total number of reads generated by the sequencing run. For the barcoded PCR enriched samples, this is the number of reads represented by a given barcode.

Conversely, each of the native samples was used to generate an independent, single‐sample genome library. The total number of reads per sample was significantly higher, with the smallest result yielding 175 492 reads. However, only a few hundred reads actually mapped to the mtGenome due to the substantially greater concentration of nuclear fragments. The average depth of coverage across the mtGenome ranged from 15 to 118x, with the lowest coverage for a single base in any given native sample being 8x (Table 1). While this may seem “insufficient”, the sequencing results for this native sample, sample 449, were equally informative/accurate as its counterpart generated from an amplicon‐enriched library with an average coverage of 334x.

### 3.2 Amplicon versus native sequence concordance

The primary goal of this project was to determine if it was possible to generate usable mtDNA sequences with an enrichment‐free approach. This was achieved by comparing amplified mtDNA sequences to native sequences extracted from whole genome sequencing libraries and assessing them for concordance. If the processes proved comparable, then it is feasible that one could substitute the faster, cheaper, and easier enrichment‐free method for the more common PCR‐based sequencing protocols.

Across the nine samples (including HL60 control) sequenced, the average number of differences between the amplified and the native consensus sequences was 102.66 differences across the 16 569 base mtGenome. The libraries using the two approaches were 99.38% concordant (Table 2). Overall, these results provide two conclusions: 1) It is possible to generate comparable results using a PCR‐free method of mitochondrial DNA sequencing, and 2) The amount of variation identified between the two sequencing methods falls within the observed error rate boundaries of the ONT sequencing technologies.

**Table 2:** Number of nucleotide differences observed and percent concordance between mitochondrial DNA sequences generated from native libraries and PCR‐amplified libraries

Sample	Number of differences (out of 16569 nt)	Percent concordance
HL60	77	99.54%
101	59	99.64%
102	105	99.36%
103	65	99.61%
433	89	99.46%
441	147	99.11%
442	118	99.28%
449	165	99.00%
459	99	99.40%
**Average**	**102.66**	**99.38%**

**Figure 1:**
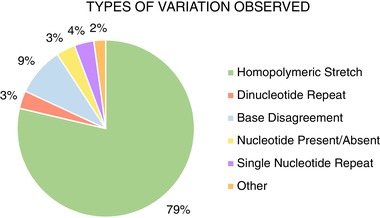
The types of differences observed between mitochondrial DNA sequences generated from native libraries and PCR‐amplified libraries were classified. Color coding is as follows: Green represents errors in a homopolymeric stretch (single nucleotide repeated 3 or more times. Example: CCCCC. Blue represents a disagreement between the base called from one strand to another. Example A/T. Red represents errors in a dinucleotide repeat region (Two nucleotides repeated at least twice. Example: ATAT). Yellow a single nucleotide outside of homopolymer regions present in one consensus sequence but not the other. Purple represents a single nucleotide repeat error (A single nucleotide repeated twice in one sequence but only once in the other. Example: GN/GG). Orange represents other errors, including a misalignment before or after a homopolymeric stretch, or a single varying nucleotide located within a homopolymeric stretch that was misaligned. Examples: AAATTT/AAAATT; AAAATAAAA/AAATAAAAA.

The differences observed were further assessed to determine the types of variations that were occurring during sequencing (Fig. 1 and Supporting Information Table 1). By and large, the majority of inconsistencies occurred within homopolymeric stretches, an issue common across sequencing platforms 63, 64. Historically, length heteroplasmy observed in homopolymeric regions from Sanger‐based sequencing for mtDNA data in forensic cases has been ignored due to the recognized ambiguity it introduced that could only be effectively quantified via cloning 65, 66, 67 or directly through electrospray ionization mass spectrometry (ESI‐MS) 68. There are two standard challenges homopolymeric stretches pose in current synthesis‐based MPS analyses: (i) The difficulty in analysis algorithms’ ability to accurately align the assemblage of reads generated by these processes 69, 70 and (ii) The chemistry‐based sequencing errors the synthesis processes induce 71, 72. Several studies have reported that sequencing platforms begin to experience difficulties with homopolymeric stretches beyond 7–9 consecutive bases 63, 64, 65. Just et al. address the complexity of heteroplasmy interpretations for both point occurrences in complex sequence regions, as well as length‐based heteroplasmy, typically observed in homopolymer stretches. These authors stress that interpretational difficulties stem from sources including the chemistry, alignment algorithms, reference bias, and parameter thresholds set by the user for data retention 63. O'Donnell et al. 73 modeled the effect that multiple identical nucleotides exceeding the k‐mer capacity of a nanopore would have on sequence accuracy in these regions. Their projections, made prior to the commercialization of a nanopore‐based sequencing platform, aligned well with the outcomes we describe below in sequencing through homopolymer regions of the mtGenome.

In this study, 79 percent of the observed differences (727 of the 924 total) consisted of discrepancies between number of bases within homopolymeric stretches, defined here by a single base being repeated three or more times in a row. Unlike other platforms, this platform struggles to separate the electrical signal into individual bases when a string of identical bases/signal translocate through the nanopore, as was postulated by O'Donnell et al. 73. Unlike other platforms, however, there are no downstream errors in alignment induced beyond the homopolymeric region. As expected, there does not appear to be a high degree of error with dinucleotide repeats, e.g., when two different adjacent bases are repeated at least twice in tandem, (compromising only 3.2%, or 30, of all observed differences). This finding indicates that the incorporation of any variable signal, e.g., the presence a hetero‐nucleotide polymer string within the k‐mer passing through the nanopore, is sufficient to prevent homopolymeric errors to be induced through signal capture during strand translocation. The reduction in homopolymer and dinucleotide repeat errors in nanopore sequencing is significant in instances where mitochondrial or nuclear microsatellite instability is suspected, as frequently described in cancer testing 74, 75, 76, 77.

Nucleotide disagreements, a difference in the base present at a single position from one sequence to the other, were observed in 9% of the discrepancies (82 of 924 occurrences). Instances in which an additional, non‐identical base was observed in one consensus sequence but not the other were detected 32 times (3.5% of the errors), and single nucleotide repeats, the same base repeated twice in one sequence and once in the other, occurred 33 times (3.6% of the errors). This fairly even distribution of variants implies that, aside from homopolymeric stretches, the types of inconsistencies or errors observed with the MinION sequencer are random. A tabulation of the observed differences can be found in Supporting Information Table 1.

A single sample, sample 449, exhibited 51 of the 82 observed base disagreements, indicating that there were a combined total of only 31 base disagreements across the other eight samples sequenced. DNA from sample 449, along with several others, were extracted from frozen blood samples up to 10 years old. DNA degradation is common in older stored samples, and often consists of cytosine bases undergoing deamination to uracil, which would be detected as a thymine in DNA sequencing that incorporates PCR enrichment or synthesis 78, 79, 80. It would be expected that the standard basecaller algorithms used with MinION data would recognize the uracil base as a thiamine as well, unless alternate basecalling rules were developed to specifically identify these base modifications. Given the numerous C/T discrepancies randomly detected in both the PCR‐enriched and native consensus sequences for sample 449, it is likely that our observations for this sample are originating from cytosine deamination.

### 3.3 Enriched versus native sequencing accuracy

The control sample HL60 was utilized to assess the accuracy of the ONT sequencing. Overall, the native sequence produced was concordant with the known HL60 sequence at 16 404 positions out of 16 569, resulting in an error rate of approximately 1.00%. The PCR‐enriched consensus sequence for HL60 was concordant with the reference sequence at 16 418 of the 16 569 positions, indicating the error rate of the enriched samples was 0.91% (Table 2). This error rate is consistent with a rate of discordance (0.62%) between the amplified and native samples outlined in section 3.1, indicating that our observed error rate of the system is less than 1%. A record of the locations and types of errors observed can be found in Supporting Information Table 2. These findings are of particular importance considering the common misconception that the Nanopore sequencing system is highly error‐prone. While this may have been true in previous years (73), improvements in the library preparation kits, flowcells and base recognition software have been successful in grossly reducing the error‐frequency of the system, as observed by Jain et al. 81.

### 3.4 Effectiveness of ONT's homopolymeric correction algorithm

Sequencing data generated for the control sample HL60 were re‐basecalled using Albacore version 1.1, which included a homopolymeric correction algorithm. To determine the effectiveness of the new algorithm, newly generated consensus sequences were aligned to the NIST‐certified HL60 sequence and compared to the original sequence generated for HL60.

The homopolymeric correction algorithm correctly called an additional 102 positions of the 165 miscalled bases identified initially from native genome sequencing, decreasing the error rate from 1.00 to 0.38%. Similarly, 101 of the 151 errors identified within the amplicon‐ based HL60 consensus sequence were corrected, bringing the error rate of the system down from 0.91 to 0.30% (Table 3). These results indicate that the new algorithm was effective in reducing errors, particularly in homopolymeric regions and was also successful in correctly identifying other bases that were originally miscalled. The results we obtained with the current iteration of homopolymer‐correcting basecaller software are consistent with that described by Jain et al. 81 when employing MinION sequencing coupled with short‐read Illumina data to generate the full autosomal human genome. Supporting Information Table 2 outlines the positions and types of errors that were corrected, as well as those errors that remained following re‐basecalling.

**Table 3:** Evaluation of efficiency of homopolymeric correction algorithm

	HL60 native		HL60 enriched	
	Original	Corrected	Original	Corrected
Number of differences (out of 16 569 nt)	165	63	151	50
Percent concordance	99.00%	99.62%	99.09%	99.70%
Error rate	1.00%	0.38%	0.91%	0.30%

The control sample HL60 was rebasecalled using ONT's new homopolymeric algorithm, and consensus sequences were compared to the reference. Overall, 102 incorrectly called nucleotides(nt) from the native sequencing were corrected, and 101 from the enriched sequencing were corrected. In each case, this improved the accuracy of the sequencing platform by approximately 66%.

## 4 Concluding remarks

Since their introduction, single molecule sequencing approaches have been challenged with regard to the potential accuracy of data and observed error rates. These systems, including the ONT MinION, have been in a constant state of evolution as technological advances in chemistries, nanoengineering, and software development continue to be adopted. Given the quality of data recently generated in numerous studies, including this study, the stigma of high error and low accuracy attributed to nanopore sequencing need to be reconsidered. The ability to *de novo* sequence genomic DNA, as well as simultaneously capture nucleotide modifications such as methylation, in a rapid and cost‐effective manner can play significant roles in genomics, epigenomics, biomedical research and clinical studies 82, 83. Although native DNA sequencing cannot compete in terms of read count and lower error rates with the current NGS platforms, significant improvements to nanopore sequencing technologies coupled to homopolymer correction algorithms and long read alignment are increasing the base calling accuracy and ability to characterize structural variants of nanopore‐generated sequences 84. For studies relying on limited samples, enrichment methods will still prove to be a vital resource in generating results that would otherwise be unlikely, and these approaches are equally amenable to sequencing on a nanopore system in multiplexed barcoded/indexed pools as they would be on current NGS platforms. The higher depth of coverage provided by enrichment plays an equivalent role across sequencing platforms in identifying and reducing chemistry‐ driven errors from final consensus sequences. However, a native genomic DNA approach can have significant advantages in field‐based applications for conservation genetics, genetic screening for mitochondrial disease, and forensic mass fatality identification efforts when the sample quality or quantity is not an impediment.

## Supporting information

Supporting Information
