# Supplementary material for: Nanopore sequencing: An enrichment‐free alternative to mitochondrial DNA sequencing
Source: Electrophoresis. 2018 Dec 13;40(2):272–80. doi: 10.1002/elps.201800083 (PMC6590251; doi:10.1002/elps.201800083)
Supplement: Supplementary file 1 — Supporting Information [file ELPS-40-272-s001.docx]

Supplementary Table 1. A tabulation of the position and type of observed variations between the amplified and native consensus sequences when sequenced on the Oxford Nanopore MinION device. Sequences were aligned in Geneious using a map to reference alignment with rCRS. Color coding is as follows: Green represents errors in a homopolymeric stretch (single nucleotide repeated 3 or more times. Example: CCCCC). Blue represents a disagreement between the base called from one strand to another. (Example A/T). Red represents errors in a dinucleotide repeat region (two nucleotides repeated at least twice. Example: ATAT). Yellow represents a single nucleotide outside of homopolymer regions present in one consensus sequence but not the other. Purple represents a single nucleotide repeat error (A single nucleotide repeated twice in one sequence but only once in the other. Example: GN/GG). Orange represents other errors, including a misalignment before or after a homopolymeric stretch, or a single varying nucleotide located within a homopolymeric stretch that was misaligned. Examples: AAATTT/AAAATT; AAAATAAAA/AAATAAAAA.

| Position | Variant (Enriched/Native) | HL60 | 101 | 102 | 103 | 433 | 441 | 442 | 449 | 459 |
| --- | --- | --- | --- | --- | --- | --- | --- | --- | --- | --- |
| 36 | G/- |  | ***** |  |  |  |  |  |  | ***** |
| 71 | G/- |  | ***** | ***** |  | ***** |  |  |  | ***** |
| 72 | T/- |  |  |  |  | ***** |  |  |  |  |
| 73 | G/- |  |  |  |  | ***** |  |  |  |  |
| 143 | G/A |  |  |  |  |  | ***** |  |  |  |
| 146 | T/C |  |  |  |  |  | ***** |  |  |  |
| 150 | C/T | ***** |  |  |  |  |  |  |  |  |
| 242 | C/T |  | ***** |  |  |  |  |  |  |  |
| 263 | G/A |  |  |  |  |  |  |  | ***** |  |
| 285 | T/C |  |  |  |  |  |  |  | ***** |  |
| 291 | A/- |  |  |  |  | ***** | ***** | ***** | ***** | ***** |
| 309 | C/- |  | ***** | ***** |  | ***** | ***** | ***** |  |  |
| 362 | A/- |  | ***** | ***** | ***** |  | ***** | ***** | ***** | ***** |
| 372 | C/- |  |  | ***** |  |  | ***** |  |  |  |
| 386 | G/A |  |  |  |  |  |  |  | ***** |  |
| 409 | T/A |  | ***** |  |  |  |  |  |  |  |
| 411 | G/- |  |  |  |  |  |  |  | ***** |  |
| 425 | T/- | ***** |  |  |  |  | ***** |  |  |  |
| 439 | C/- |  |  |  |  |  | ***** |  |  |  |
| 460 | C/- |  | ***** |  |  | ***** |  | ***** |  |  |
| 464 | C/- |  |  |  |  |  | ***** |  |  |  |
| 498 | C/T |  |  |  |  |  |  |  | ***** |  |
| 499 | -/C |  |  |  |  |  |  |  | ***** |  |
| 522 | -/A |  |  |  |  |  |  |  | ***** |  |
| 522 | A/- |  |  |  |  |  |  |  |  | ***** |
| 523 | -/C |  |  |  |  |  |  |  | ***** |  |
| 523 | C/- |  |  |  |  |  |  |  |  | ***** |
| 524 | -/A |  |  |  |  |  |  |  | ***** |  |
| 524 | A/- |  |  |  |  |  |  |  |  | ***** |
| 525 | -/C | ***** |  |  |  |  |  |  | ***** |  |
| 525 | C/- |  |  |  |  |  |  |  |  | ***** |
| 574 | -/C | ***** |  |  |  |  |  |  |  |  |
| 574 | C/- |  |  | ***** |  | ***** | ***** |  | ***** | ***** |
| 616 | A/- |  |  |  |  |  |  |  |  | ***** |
| 651 | C/- |  |  | ***** |  |  |  |  |  |  |
| 727 | C/- |  |  |  |  |  |  | ***** |  |  |
| 748 | -/A |  |  |  |  |  |  |  |  |  |
| 751 | G/- |  |  |  |  |  |  |  | ***** |  |
| 751 | G/A |  |  |  |  | ***** |  |  |  |  |
| 795 | T/C |  |  |  |  |  |  |  | ***** |  |
| 807 | C/- | ***** | ***** | ***** |  |  | ***** | ***** | ***** | ***** |
| 812 | G/- |  |  |  |  | ***** |  |  |  |  |
| 960 | T/C |  |  |  |  | ***** |  |  |  |  |
| 960 | C/- |  |  | ***** | ***** |  |  | ***** |  |  |
| 961 | C/- |  |  |  |  |  | ***** |  |  |  |
| 962 | C/T |  |  |  |  | ***** |  |  |  |  |
| 966 | C/- | ***** |  | ***** |  |  | ***** | ***** |  | ***** |
| 998 | A/- | ***** |  |  |  |  |  | ***** |  | ***** |
| 1049 | C/- |  |  |  |  |  |  |  | ***** | ***** |
| 1163 | A/- |  |  |  |  |  | ***** |  |  |  |
| 1169 | A/- |  |  | ***** |  |  |  |  |  |  |
| 1169 | -/A | ***** |  |  |  |  |  |  |  |  |
| 1193 | C/- |  |  |  |  |  |  |  |  | ***** |
| 1227 | C/T |  |  |  |  |  |  |  | ***** |  |
| 1313 | C/- |  |  |  |  | ***** |  |  |  |  |
| 1338 | T/- |  |  |  |  |  |  |  | ***** |  |
| 1339 | A/- |  |  |  |  |  |  |  | ***** |  |
| 1385 | A/- |  |  |  |  |  |  |  |  | ***** |
| 1515 | A/- |  |  |  |  |  | ***** |  |  |  |
| 1515 | -/A | ***** |  |  |  |  |  |  |  |  |
| 1535 | C/- |  |  | ***** |  |  |  | ***** |  |  |
| 1615 | -/T | ***** |  |  |  |  |  |  |  |  |
| 1615 | T/- |  |  |  |  |  |  | ***** |  |  |
| 1655 | T/- |  |  |  |  |  |  | ***** |  | ***** |
| 1794 | G/- |  |  | ***** |  |  |  |  |  | ***** |
| 1806 | -/A |  | ***** |  |  |  |  |  |  |  |
| 1806 | A/- |  |  | ***** | ***** | ***** | ***** | ***** | ***** |  |
| 1812 | A/G |  |  |  |  |  |  |  | ***** |  |
| 1841 | C/- |  |  |  |  |  |  | ***** | ***** |  |
| 1906 | -/C | ***** |  |  |  | ***** |  |  |  | ***** |
| 1939 | A/- |  |  | ***** |  |  | ***** | ***** |  |  |
| 1971 | G/- |  |  |  |  | ***** |  |  |  |  |
| 2058 | T/A |  |  |  |  |  | ***** |  |  |  |
| 2070 | C/T |  |  |  |  |  | ***** |  |  |  |
| 2071 | T/C |  |  |  |  |  | ***** |  |  |  |
| 2072 | C/T |  |  |  |  |  | ***** |  |  |  |
| 2076 | A/T |  |  |  |  |  | ***** |  |  |  |
| 2077 | T/C |  |  |  |  |  | ***** |  |  |  |
| 2080 | -/C | ***** |  |  |  |  |  | ***** |  |  |
| 2135 | A/- |  |  |  |  |  |  |  |  | ***** |
| 2143 | A/- |  |  |  |  |  |  |  |  | ***** |
| 2157 | A/- |  |  |  |  |  | ***** | ***** |  | ***** |
| 2182 | A/- |  |  |  |  |  |  | ***** |  |  |
| 2219 | T/C |  |  |  |  |  |  |  | ***** |  |
| 2232 | A/- |  |  |  |  |  |  |  |  | ***** |
| 2419 | C/- |  |  |  |  |  | ***** |  |  |  |
| 2471 | A/- |  |  |  |  |  | ***** |  |  |  |
| 2492 | C/- |  |  | ***** | ***** |  |  |  |  | ***** |
| 2492 | -/C | ***** |  |  |  |  |  |  |  |  |
| 2508 | A/- |  | ***** | ***** |  | ***** |  | ***** | ***** | ***** |
| 2509 | A/- |  |  |  |  |  | ***** |  |  |  |
| 2648 | G/- |  |  | ***** |  |  |  |  |  |  |
| 2649 | G/- |  |  |  |  |  | ***** |  |  |  |
| 2806 | A/- | ***** | ***** |  |  | ***** | ***** | ***** | ***** | ***** |
| 2827 | G/- |  |  |  |  |  |  |  | ***** |  |
| 2837 | T/C |  |  |  |  |  |  |  | ***** |  |
| 2935 | G/- | ***** |  |  |  |  |  |  | ***** |  |
| 3010 | C/T |  |  |  |  |  |  |  | ***** |  |
| 3035 | T/C |  |  |  |  |  |  |  | ***** |  |
| 3121 | C/- |  |  | ***** |  |  |  |  |  |  |
| 3138 | G/- |  |  |  |  |  | ***** |  |  |  |
| 3194 | T/- |  |  | ***** |  |  |  |  |  |  |
| 3375 | C/T |  |  |  |  |  |  |  | ***** |  |
| 3385 | A/- | ***** | ***** |  |  | ***** |  |  |  | ***** |
| 3386 | A/- |  |  |  |  |  | ***** | ***** |  |  |
| 3411 | A/- |  |  |  | ***** |  | ***** |  |  |  |
| 3432 | C/- |  | ***** | ***** |  |  | ***** | ***** |  |  |
| 3480 | A/G |  |  |  |  |  |  |  | ***** |  |
| 3487 | C/- |  |  | ***** |  | ***** | ***** | ***** |  |  |
| 3487 | -/C | ***** |  |  |  |  |  |  |  |  |
| 3488 | C/- |  |  |  |  |  | ***** |  |  |  |
| 3525 | C/T |  |  |  |  |  |  |  | ***** |  |
| 3569 | C/- |  |  |  |  |  |  | ***** |  |  |
| 3571 | -/C | ***** |  |  |  |  |  |  |  |  |
| 3578 | C/- |  |  |  |  |  | ***** | ***** |  |  |
| 3591 | A/G |  |  |  |  |  |  |  | ***** |  |
| 3642 | C/T |  |  |  |  |  |  |  | ***** |  |
| 3666 | G/- |  |  |  |  |  |  |  | ***** |  |
| 3742 | C/- |  |  | ***** |  |  |  |  |  |  |
| 3781 | T/- |  |  |  |  |  |  |  | ***** |  |
| 3963 | -/C |  |  |  |  |  |  |  | ***** |  |
| 3963 | C/- |  |  | ***** |  |  |  |  |  |  |
| 4015 | C/- |  |  |  |  |  |  | ***** |  |  |
| 4060 | C/- |  |  |  |  |  |  | ***** |  |  |
| 4063 | C/- |  |  |  |  |  | ***** |  |  |  |
| 4111 | C/- | ***** |  |  |  |  |  |  |  | ***** |
| 4113 | C/- |  |  |  |  |  | ***** |  |  |  |
| 4141 | C/- |  |  |  |  | ***** |  | ***** | ***** |  |
| 4141 | -/C |  |  |  |  |  |  |  |  | ***** |
| 4143 | C/- |  |  |  |  |  | ***** |  |  |  |
| 4152 | C/T |  |  |  |  |  |  |  | ***** |  |
| 4281 | -/A |  |  |  |  | ***** |  |  | ***** |  |
| 4281 | A/- |  |  |  |  |  |  | ***** |  |  |
| 4314 | T/A |  |  |  |  |  |  |  | ***** |  |
| 4317 | A/- |  |  |  |  |  |  |  | ***** |  |
| 4318 | C/- |  |  |  |  |  |  |  | ***** |  |
| 4322 | -/C |  |  |  |  |  |  |  | ***** |  |
| 4431 | G/- |  |  |  |  |  | ***** |  |  |  |
| 4439 | C/- |  |  |  | ***** |  |  |  |  |  |
| 4446 | A/- |  |  |  |  |  | ***** |  |  |  |
| 4458 | C/- |  |  |  |  | ***** |  |  |  |  |
| 4463 | C/T |  |  |  |  |  |  |  | ***** |  |
| 4490 | C/T |  |  |  |  |  |  |  | ***** |  |
| 4552 | T/- |  |  |  |  |  |  |  | ***** |  |
| 4650 | C/- |  |  |  |  | ***** |  | ***** | ***** |  |
| 4664 | C/T |  |  |  |  |  |  |  | ***** |  |
| 4789 | G/- |  |  |  |  |  |  | ***** |  |  |
| 4801 | C/- |  |  |  |  |  | ***** |  |  |  |
| 4802 | T/- |  |  |  |  |  |  |  | ***** |  |
| 4837 | -/C |  |  |  |  |  |  |  | ***** | ***** |
| 4874 | A/- |  | ***** | ***** |  | ***** |  | ***** |  |  |
| 4874 | C/- |  |  |  |  |  |  |  |  | ***** |
| 4883 | C/- | ***** | ***** | ***** | ***** | ***** |  | ***** | ***** | ***** |
| 4885 | C/- |  |  |  |  |  | ***** |  |  |  |
| 4910 | -/C |  | ***** | ***** | ***** |  | ***** | ***** | ***** | ***** |
| 4936 | C/- |  |  |  |  |  |  |  |  | ***** |
| 5211 | C/- |  |  |  |  |  |  |  |  | ***** |
| 5213 | C/- |  |  |  |  |  | ***** | ***** |  |  |
| 5213 | T/- |  |  |  |  |  |  |  |  |  |
| 5220 | C/- |  |  | ***** |  |  |  | ***** |  | ***** |
| 5226 | G/- |  |  |  |  |  | ***** |  |  |  |
| 5236 | C/T | ***** |  |  |  |  |  |  |  |  |
| 5237 | G/- |  |  |  |  |  |  |  |  | ***** |
| 5237 | G/A |  |  |  |  |  |  |  | ***** |  |
| 5237 | G/- |  |  | ***** |  |  |  |  |  |  |
| 5238 | G/- |  |  | ***** |  |  |  |  |  |  |
| 5251 | T/- |  | ***** | ***** | ***** | ***** |  | ***** |  | ***** |
| 5286 | A/- |  |  |  |  |  |  | ***** |  | ***** |
| 5328 | C/- |  |  |  |  | ***** |  |  |  |  |
| 5387 | C/- |  |  | ***** |  | ***** |  |  |  |  |
| 5389 | C/- |  |  |  |  |  | ***** |  |  |  |
| 5406 | A/- |  | ***** | ***** | ***** | ***** |  | ***** | ***** | ***** |
| 5408 | A/- |  |  |  |  |  | ***** |  |  |  |
| 5413 | A/- |  |  |  |  |  | ***** |  |  |  |
| 5471 | G/- |  |  |  |  |  |  |  |  | ***** |
| 5495 | T/- | ***** |  |  |  |  |  |  |  | ***** |
| 5497 | T/- |  |  |  |  |  | ***** |  |  |  |
| 5606 | C/- |  |  |  |  |  | ***** |  |  |  |
| 5644 | A/- |  |  |  | ***** |  |  |  |  |  |
| 5718 | G/C |  |  |  |  |  |  |  |  |  |
| 5751 | A/- |  |  |  | ***** |  |  | ***** | ***** |  |
| 5752 | A/- |  | ***** | ***** |  |  |  |  |  |  |
| 5753 | G/A | ***** |  |  |  |  |  |  |  |  |
| 5754 | A/- |  |  |  |  |  | ***** |  |  |  |
| 5755 | C/G | ***** |  |  |  |  |  |  |  |  |
| 5756 | G/C | ***** |  |  |  |  |  |  |  |  |
| 5760 | G/- |  |  |  |  |  | ***** |  |  |  |
| 5773 | G/A |  |  |  |  |  |  |  | ***** |  |
| 5783 | A/G |  |  |  |  |  |  |  |  |  |
| 5823 | G/A |  |  |  |  |  | ***** |  |  |  |
| 5847 | C/- |  |  | ***** |  |  |  |  |  |  |
| 5849 | C/- |  |  |  |  |  | ***** |  |  |  |
| 5890 | T/- |  |  |  |  |  | ***** |  |  |  |
| 5899 | C/- |  |  | ***** | ***** |  |  | ***** | ***** |  |
| 5901 | C/- |  |  |  |  |  | ***** |  |  |  |
| 6158 | C/- |  |  |  |  |  | ***** |  |  |  |
| 6172 | C/T |  |  |  |  |  |  | ***** | ***** | ***** |
| 6173 | C/- |  | ***** | ***** |  | ***** | ***** | ***** |  |  |
| 6189 | -/C | ***** |  |  |  | ***** |  |  | ***** |  |
| 6224 | C/- |  |  |  |  |  |  | ***** |  |  |
| 6225 | T/- | ***** |  |  |  |  |  |  |  |  |
| 6296 | C/- |  |  | ***** |  |  |  |  |  |  |
| 6373 | C/- |  |  |  |  |  | ***** |  |  |  |
| 6384 | G/- | ***** |  |  | ***** |  | ***** | ***** | ***** |  |
| 6450 | -/C | ***** | ***** |  |  |  |  |  | ***** |  |
| 6509 | -/A |  |  |  |  |  |  |  |  | ***** |
| 6616 | T/- | ***** |  | ***** |  |  | ***** | ***** | ***** |  |
| 6696 | A/- |  |  |  |  |  |  | ***** |  | ***** |
| 6755 | G/- |  |  |  |  |  |  | ***** |  |  |
| 6776 | T/C |  | ***** |  |  |  |  |  |  |  |
| 6930 | G/A |  |  | ***** |  | ***** |  |  |  |  |
| 6932 | A/- |  |  |  |  | ***** |  |  |  |  |
| 6946 | T/- | ***** |  |  |  |  |  | ***** | ***** |  |
| 7098 | -/C |  |  | ***** |  |  |  |  |  |  |
| 7215 | C/T |  |  |  |  |  |  |  | ***** |  |
| 7227 | G/- |  |  |  |  |  |  |  | ***** |  |
| 7367 | C/- |  |  |  | ***** |  |  | ***** |  |  |
| 7369 | C/- |  |  |  |  |  | ***** |  |  |  |
| 7401 | C/- |  |  |  |  |  |  | ***** |  |  |
| 7402 | -/C | ***** |  |  |  |  |  |  |  |  |
| 7404 | C/- |  |  |  |  |  | ***** |  |  |  |
| 7421 | C/- |  |  |  |  |  |  |  |  |  |
| 7451 | A/- | ***** |  |  | ***** |  |  |  | ***** | ***** |
| 7452 | -/A |  |  |  |  | ***** | ***** |  |  |  |
| 7452 | A/- |  | ***** | ***** |  |  |  | ***** |  |  |
| 7470 | C/- |  |  |  | ***** |  |  |  |  |  |
| 7471 | -/C |  |  |  |  |  |  |  | ***** |  |
| 7471 | C/- |  |  | ***** |  |  |  |  |  |  |
| 7473 | C/- |  |  |  |  |  | ***** |  |  |  |
| 7513 | T/- |  | ***** | ***** | ***** | ***** | ***** |  | ***** | ***** |
| 7532 | A/- |  |  |  | ***** |  |  |  |  |  |
| 7534 | A/- |  |  |  |  |  | ***** |  |  |  |
| 7575 | T/- |  |  |  |  |  |  |  | ***** |  |
| 7576 | A/- | ***** |  |  |  |  |  |  |  | ***** |
| 7581 | C/T |  |  |  |  |  |  |  | ***** |  |
| 7629 | -/C |  | ***** | ***** | ***** |  |  |  | ***** |  |
| 7654 | T/- |  |  |  |  |  |  |  | ***** |  |
| 7864 | -/C |  |  |  | ***** |  |  |  |  | ***** |
| 7866 | C/- |  |  |  |  |  | ***** |  |  |  |
| 7868 | -/C |  |  |  |  | ***** |  |  |  |  |
| 8032 | C/- |  | ***** | ***** | ***** |  | ***** | ***** | ***** | ***** |
| 8085 | C/- |  |  |  |  |  | ***** |  |  |  |
| 8095 | A/T |  |  |  |  |  |  |  | ***** |  |
| 8099 | A/- |  | ***** | ***** | ***** | ***** | ***** | ***** |  | ***** |
| 8115 | C/- |  |  |  |  |  | ***** |  |  |  |
| 8156 | G/- |  |  | ***** |  | ***** |  |  |  | ***** |
| 8165 | G/T |  |  |  |  |  |  |  | ***** |  |
| 8231 | -/C |  |  |  |  |  |  |  | ***** |  |
| 8237 | A/- |  | ***** | ***** | ***** | ***** | ***** | ***** | ***** |  |
| 8251 | G/- |  |  |  |  |  |  | ***** |  |  |
| 8275 | C/- |  |  |  |  |  |  | ***** |  |  |
| 8374 | C/- |  |  |  |  |  | ***** |  |  |  |
| 8388 | T/C |  |  |  |  |  |  |  | ***** |  |
| 8410 | C/- | ***** |  | ***** | ***** |  |  | ***** | ***** |  |
| 8447 | A/- | ***** |  |  |  | ***** | ***** | ***** | ***** | ***** |
| 8476 | C/- | ***** |  | ***** |  |  | ***** | ***** |  |  |
| 8495 | A/- | ***** | ***** | ***** | ***** | ***** | ***** | ***** | ***** | ***** |
| 8562 | C/- | ***** |  | ***** |  |  |  |  | ***** |  |
| 8608 | C/- |  |  |  |  |  |  |  | ***** |  |
| 8609 | -/C | ***** |  |  |  |  |  | ***** | ***** | ***** |
| 8760 | T/- | ***** |  |  |  |  |  |  | ***** | ***** |
| 8847 | C/- |  |  | ***** |  |  |  |  |  |  |
| 8847 | -/C |  |  |  |  |  |  | ***** | ***** |  |
| 8849 | -/C |  |  |  |  |  | ***** |  |  |  |
| 8894 | A/T |  |  |  |  |  |  |  |  | ***** |
| 8894 | A/- |  | ***** |  | ***** | ***** |  | ***** | ***** |  |
| 8895 | G/T |  |  |  |  |  |  |  |  | ***** |
| 8896 | C/G |  |  |  |  |  |  |  |  | ***** |
| 8908 | C/T |  | ***** |  | ***** | ***** |  |  |  | ***** |
| 8935 | C/- |  |  | ***** |  |  | ***** |  |  |  |
| 9012 | T/C |  |  |  |  |  |  |  | ***** |  |
| 9055 | G/A |  |  |  |  |  |  |  | ***** |  |
| 9087 | -/C |  | ***** | ***** | ***** | ***** | ***** | ***** | ***** |  |
| 9258 | C/- |  |  |  |  |  |  |  |  | ***** |
| 9258 | -/C |  |  | ***** |  |  |  |  |  |  |
| 9417 | C/- |  |  |  |  |  |  |  |  | ***** |
| 9475 | A/- |  |  |  |  |  | ***** |  |  |  |
| 9486 | T/- |  |  |  |  |  | ***** |  |  |  |
| 9499 | T/- |  |  | ***** |  |  | ***** | ***** |  | ***** |
| 9510 | T/- | ***** |  |  |  |  |  |  |  |  |
| 9531 | C/- |  |  |  |  |  | ***** |  |  |  |
| 9537 | -/C |  |  |  |  |  |  |  | ***** |  |
| 9537 | C/- | ***** |  |  |  | ***** |  |  |  |  |
| 9559 | C/- |  | ***** | ***** |  | ***** | ***** | ***** |  |  |
| 9575 | A/G |  |  |  |  |  |  |  | ***** |  |
| 9585 | -/C | ***** |  |  |  | ***** | ***** | ***** | ***** | ***** |
| 9667 | A/- | ***** |  |  |  |  |  |  |  |  |
| 9669 | A/- |  |  |  |  |  | ***** |  |  |  |
| 9731 | C/- |  |  |  |  |  | ***** |  |  |  |
| 9761 | -/C |  | ***** |  | ***** | ***** |  | ***** |  |  |
| 9998 | T/- |  |  |  |  |  |  |  |  | ***** |
| 10023 | -/C |  | ***** | ***** |  | ***** | ***** | ***** | ***** | ***** |
| 10052 | A/- | ***** |  |  |  |  |  | ***** | ***** | ***** |
| 10053 | A/- |  | ***** | ***** |  |  | ***** |  |  |  |
| 10067 | C/A |  |  |  |  |  |  |  | ***** |  |
| 10092 | C/- |  |  |  |  |  |  | ***** |  |  |
| 10165 | -/C |  | ***** |  |  | ***** |  |  | ***** |  |
| 10196 | -/C |  |  |  |  |  |  |  | ***** |  |
| 10238 | T/C |  |  |  |  |  |  |  | ***** |  |
| 10276 | T/- |  |  |  |  |  |  | ***** | ***** |  |
| 10332 | C/- |  | ***** | ***** |  |  |  | ***** |  |  |
| 10385 | -/A |  |  | ***** | ***** |  |  |  | ***** | ***** |
| 10398 | A/G |  |  |  |  |  |  |  | ***** |  |
| 10476 | -/C |  |  |  |  |  |  |  | ***** |  |
| 10550 | A/G |  |  |  |  |  |  |  | ***** |  |
| 10629 | -/C |  |  |  |  | ***** |  |  |  |  |
| 10629 | C/- | ***** | ***** | ***** |  |  |  |  | ***** |  |
| 10640 | T/C |  |  |  |  |  |  | ***** |  |  |
| 10818 | A/- |  |  |  | ***** |  |  |  |  |  |
| 10819 | A/- | ***** |  | ***** |  | ***** | ***** |  |  |  |
| 10872 | C/- | ***** |  |  |  |  |  |  |  |  |
| 10885 | -/T |  |  |  |  |  |  |  | ***** |  |
| 10920 | C/- |  |  | ***** | ***** |  |  | ***** |  |  |
| 10920 | -/C | ***** |  |  |  |  | ***** |  |  | ***** |
| 10951 | C/- |  |  |  | ***** |  |  | ***** |  |  |
| 10952 | C/- |  | ***** | ***** |  | ***** | ***** |  |  |  |
| 10982 | -/C | ***** |  | ***** |  |  |  |  | ***** | ***** |
| 11025 | T/C |  |  |  |  |  |  |  | ***** |  |
| 11063 | C/- |  |  |  |  | ***** |  | ***** | ***** |  |
| 11162 | C/T |  |  |  |  |  |  | ***** |  |  |
| 11231 | T/C |  |  |  |  | ***** |  |  | ***** |  |
| 11231 | -/C | ***** |  |  |  |  |  |  |  |  |
| 11233 | -/T |  |  |  |  | ***** |  |  | ***** |  |
| 11234 | -/C |  |  |  |  | ***** |  |  | ***** |  |
| 11237 | -/C |  | ***** |  |  |  |  |  |  | ***** |
| 11270 | C/- |  |  |  | ***** |  |  |  |  |  |
| 11387 | T/- |  |  |  |  |  |  |  | ***** |  |
| 11402 | T/- |  |  |  |  |  | ***** |  |  |  |
| 11409 | C/- |  |  | ***** |  |  |  |  |  |  |
| 11411 | C/- |  |  |  |  |  | ***** |  |  |  |
| 11431 | C/- | ***** |  | ***** | ***** | ***** | ***** | ***** |  | ***** |
| 11541 | C/- |  |  |  |  |  | ***** | ***** |  | ***** |
| 11716 | C/T |  |  |  |  |  | ***** |  |  |  |
| 11770 | C/T |  |  |  |  |  |  |  | ***** |  |
| 11799 | A/- |  |  |  |  |  | ***** |  |  |  |
| 11831 | T/- |  |  |  | ***** | ***** |  | ***** | ***** |  |
| 11854 | T/C |  |  |  |  |  |  |  | ***** |  |
| 11860 | C/- |  |  |  |  |  |  |  | ***** |  |
| 11872 | C/- |  |  | ***** |  |  |  |  |  |  |
| 11874 | C/- |  |  |  |  |  | ***** |  |  |  |
| 11946 | C/T |  |  |  |  |  | ***** |  |  |  |
| 11974 | T/A |  |  |  |  |  |  |  | ***** |  |
| 11975 | -/T |  |  |  |  |  |  |  | ***** |  |
| 11976 | -/A |  |  |  |  |  |  |  | ***** |  |
| 11981 | C/- |  |  | ***** |  |  |  | ***** |  |  |
| 11983 | C/- |  |  |  |  |  | ***** |  |  |  |
| 12104 | -/C |  | ***** | ***** |  |  | ***** |  |  | ***** |
| 12197 | -/C | ***** | ***** |  | ***** | ***** |  |  |  | ***** |
| 12242 | C/- | ***** |  |  |  |  |  |  |  | ***** |
| 12243 | C/- |  |  |  |  |  | ***** | ***** |  |  |
| 12262 | C/- |  |  |  |  | ***** |  |  |  |  |
| 12308 | G/A |  |  |  |  |  |  |  | ***** |  |
| 12310 | A/- |  | ***** | ***** | ***** |  | ***** | ***** |  | ***** |
| 12333 | A/- |  |  |  |  |  |  | ***** |  |  |
| 12364 | C/- |  |  | ***** | ***** |  | ***** |  |  |  |
| 12379 | -/C |  |  |  |  | ***** |  |  |  | ***** |
| 12379 | C/- | ***** | ***** | ***** |  |  | ***** |  |  |  |
| 12389 | C/- |  |  |  | ***** |  |  |  |  |  |
| 12413 | C/- |  |  | ***** |  |  |  |  |  |  |
| 12436 | C/- |  | ***** | ***** | ***** | ***** | ***** | ***** | ***** | ***** |
| 12486 | C/- |  | ***** |  | ***** | ***** | ***** | ***** |  |  |
| 12514 | G/- |  |  | ***** |  |  |  |  |  |  |
| 12568 | C/- |  |  |  |  | ***** |  |  |  |  |
| 12568 | C/- |  | ***** | ***** | ***** |  | ***** | ***** |  |  |
| 12710 | T/- |  |  |  |  |  | ***** |  |  |  |
| 12879 | C/T |  |  |  |  |  |  |  | ***** |  |
| 12892 | G/- |  |  |  |  |  | ***** |  |  |  |
| 13029 | -/C |  | ***** | ***** | ***** |  |  |  | ***** |  |
| 13037 | C/- |  |  |  |  |  |  | ***** |  |  |
| 13038 | -/C |  |  |  |  |  |  |  | ***** | ***** |
| 13056 | C/- |  |  | ***** |  | ***** |  |  |  |  |
| 13059 | C/- |  |  |  |  |  | ***** |  |  |  |
| 13104 | G/A |  |  |  |  |  |  |  | ***** |  |
| 13129 | C/- |  |  |  |  |  | ***** |  |  |  |
| 13133 | -/C | ***** |  |  |  |  |  |  |  |  |
| 13422 | G/A |  |  |  |  |  |  |  | ***** |  |
| 13434 | A/G |  |  |  |  |  |  |  | ***** |  |
| 13454 | C/- | ***** |  |  |  |  | ***** | ***** | ***** | ***** |
| 13588 | -/C |  |  |  | ***** |  |  |  | ***** |  |
| 13588 | C/- |  |  | ***** |  |  |  |  |  |  |
| 13589 | C/- | ***** |  |  |  |  |  |  |  | ***** |
| 13591 | C/- |  |  |  |  |  | ***** |  |  |  |
| 13627 | C/- |  |  | ***** |  | ***** |  |  |  |  |
| 13630 | C/- |  |  |  |  |  | ***** |  |  |  |
| 13650 | C/- | ***** | ***** | ***** | ***** | ***** |  | ***** |  |  |
| 13683 | C/- |  |  |  |  |  | ***** |  |  |  |
| 13757 | C/- |  |  |  | ***** |  |  | ***** |  | ***** |
| 13761 | C/- |  |  |  |  |  | ***** |  |  |  |
| 13808 | G/- |  |  |  |  |  | ***** |  |  |  |
| 13825 | G/A |  |  |  |  |  |  | ***** |  |  |
| 13883 | C/- |  |  |  |  |  |  | ***** | ***** | ***** |
| 13887 | C/- |  |  |  |  |  | ***** |  |  |  |
| 13915 | G/A |  |  |  |  | ***** |  |  |  |  |
| 13917 | A/- |  |  |  |  | ***** |  |  | ***** |  |
| 13927 | G/A |  |  | ***** | ***** |  |  |  |  |  |
| 13951 | -/C | ***** |  |  |  |  |  | ***** |  | ***** |
| 13956 | G/A |  |  |  |  | ***** |  |  | ***** |  |
| 13984 | C/- |  |  | ***** |  | ***** |  |  |  |  |
| 13985 | T/- |  |  |  | ***** |  |  |  |  |  |
| 13988 | C/- |  |  |  |  |  | ***** |  |  |  |
| 14014 | A/- |  |  |  |  |  | ***** |  |  |  |
| 14015 | A/- |  |  | ***** |  | ***** |  |  |  |  |
| 14019 | A/- |  |  |  |  |  | ***** |  |  |  |
| 14070 | G/A |  |  |  |  |  |  |  | ***** |  |
| 14095 | T/- |  |  |  |  |  |  |  | ***** |  |
| 14100 | C/- |  |  |  |  |  |  | ***** |  |  |
| 14101 | C/- |  |  |  |  |  |  | ***** |  |  |
| 14131 | C/- |  |  |  |  | ***** |  |  |  |  |
| 14158 | C/T |  |  |  |  |  |  | ***** | ***** |  |
| 14248 | C/- |  |  |  | ***** |  |  |  |  |  |
| 14248 | -/C | ***** | ***** |  |  |  |  |  |  | ***** |
| 14268 | C/T | ***** |  |  |  |  |  | ***** |  |  |
| 14284 | C/- |  | ***** | ***** |  |  |  | ***** |  |  |
| 14284 | -/C | ***** |  |  |  |  |  |  |  | ***** |
| 14323 | G/A |  |  |  |  | ***** |  |  |  |  |
| 14343 | C/- |  |  | ***** |  |  |  |  |  |  |
| 14413 | C/- |  |  |  |  |  |  | ***** |  |  |
| 14421 | C/- |  |  | ***** | ***** | ***** |  | ***** | ***** | ***** |
| 14425 | C/- |  |  |  |  |  | ***** |  |  |  |
| 14427 | C/T |  |  |  |  | ***** |  |  |  |  |
| 14428 | C/T |  |  |  |  |  |  | ***** |  |  |
| 14429 | C/- |  |  |  |  |  |  |  | ***** |  |
| 14429 | C/- |  |  |  |  | ***** |  |  |  |  |
| 14433 | C/- |  |  |  |  |  | ***** |  |  |  |
| 14493 | C/- |  |  |  |  | ***** |  |  |  |  |
| 14494 | -/C |  |  |  |  |  |  |  |  | ***** |
| 14510 | A/- |  |  | ***** |  |  |  |  |  |  |
| 14536 | -/C |  |  |  |  |  |  |  |  | ***** |
| 14592 | C/- |  |  | ***** | ***** | ***** |  |  | ***** |  |
| 14593 | C/- | ***** |  |  |  |  | ***** | ***** |  | ***** |
| 14611 | A/- |  |  |  | ***** |  |  | ***** |  |  |
| 14611 | -/T | ***** |  |  |  |  |  |  |  |  |
| 14614 | T/- |  |  |  |  |  | ***** |  |  |  |
| 14614 | A/- |  |  |  |  | ***** | ***** |  |  |  |
| 14615 | A/- |  |  |  |  |  | ***** |  |  |  |
| 14623 | C/- |  |  |  |  |  |  |  | ***** |  |
| 14627 | C/- |  |  |  |  |  | ***** |  |  |  |
| 14715 | A/- |  | ***** | ***** |  |  |  |  |  | ***** |
| 14719 | A/- |  |  |  |  |  | ***** |  |  |  |
| 14773 | C/- |  |  |  |  |  |  | ***** |  |  |
| 14774 | -/C |  |  |  |  |  |  |  | ***** |  |
| 14798 | T/C |  |  |  |  |  |  |  | ***** |  |
| 14812 | A/C |  |  |  |  | ***** |  |  |  |  |
| 14812 | C/- |  | ***** |  |  |  |  |  |  |  |
| 14813 | A/C |  |  |  |  | ***** |  |  |  |  |
| 14813 | C/- | ***** |  |  |  |  |  |  |  |  |
| 14816 | C/- |  |  |  |  |  | ***** |  |  |  |
| 14882 | C/A |  |  |  | ***** |  |  |  |  |  |
| 14883 | G/C |  |  |  | ***** |  |  |  |  |  |
| 14884 | A/C |  |  |  | ***** |  |  |  |  |  |
| 14889 | C/G |  |  |  | ***** |  |  |  |  |  |
| 14893 | -/A |  |  |  | ***** |  |  |  |  |  |
| 14894 | -/T |  |  |  | ***** |  |  |  |  |  |
| 15034 | A/- |  |  |  | ***** |  |  |  |  |  |
| 15043 | G/- |  |  |  |  |  |  | ***** |  |  |
| 15148 | A/G |  |  |  |  |  |  |  | ***** |  |
| 15173 | G/- |  |  | ***** |  | ***** |  |  |  |  |
| 15177 | G/- |  |  |  |  |  | ***** |  |  |  |
| 15243 | G/- |  |  | ***** |  |  |  |  |  |  |
| 15289 | T/- |  |  |  |  | ***** |  |  |  |  |
| 15359 | G/A |  |  |  |  |  | ***** |  |  |  |
| 15434 | C/- |  |  |  |  |  |  | ***** |  |  |
| 15446 | C/- |  |  |  |  |  |  | ***** | ***** |  |
| 15446 | C/- |  | ***** |  |  |  |  |  |  |  |
| 15518 | C/- |  |  | ***** |  |  |  |  |  |  |
| 15540 | -/C |  |  |  |  |  |  |  | ***** |  |
| 15540 | C/- |  |  | ***** |  |  |  |  |  |  |
| 15541 | -/C | ***** |  |  |  |  |  |  |  | ***** |
| 15545 | C/- |  |  | ***** |  | ***** | ***** | ***** |  |  |
| 15728 | C/- |  |  | ***** |  |  |  |  |  |  |
| 15787 | T/- |  |  | ***** | ***** |  |  |  | ***** |  |
| 15788 | T/- | ***** |  |  |  |  |  |  |  | ***** |
| 15861 | C/- |  |  |  |  |  | ***** |  |  |  |
| 15867 | A/- |  |  |  |  |  |  | ***** |  |  |
| 15871 | A/- |  |  |  |  |  | ***** |  |  |  |
| 15944 | T/- |  |  |  |  |  |  |  | ***** |  |
| 15945 | T/- | ***** |  |  |  |  |  | ***** |  | ***** |
| 15954 | C/A |  |  |  |  |  |  |  | ***** |  |
| 15966 | A/- |  | ***** |  |  |  |  |  |  |  |
| 16053 | G/- |  |  |  |  |  | ***** |  |  |  |
| 16093 | T/C |  | ***** |  |  |  |  |  |  |  |
| 16119 | A/G |  |  |  |  |  |  |  | ***** |  |
| 16166 | A/- | ***** | ***** | ***** |  |  |  | ***** | ***** | ***** |
| 16170 | A/- |  |  |  |  |  | ***** |  |  |  |
| 16183 | -/A |  |  |  |  |  |  |  | ***** |  |
| 16184 | -/C |  |  |  |  |  |  |  | ***** |  |
| 16188 | A/- |  |  |  |  |  | ***** |  |  |  |
| 16188 | C/- |  |  |  |  |  |  |  | ***** |  |
| 16189 | C/- |  |  |  |  |  | ***** |  |  |  |
| 16193 | C/- |  |  | ***** |  |  |  |  | ***** | ***** |
| 16224 | T/- |  |  |  |  |  |  |  | ***** |  |
| 16263 | -/C | ***** |  |  |  |  |  |  | ***** |  |
| 16311 | T/C |  |  |  |  |  |  |  | ***** |  |
| 16362 | T/- |  |  |  |  |  |  | ***** |  |  |
| 16379 | C/- |  |  |  | ***** |  |  | ***** |  |  |
| 16380 | -/C |  |  |  |  |  |  |  | ***** |  |
| 16380 | C/- |  |  | ***** |  | ***** |  |  |  |  |
| 16381 | -/C | ***** |  |  |  |  |  |  |  | ***** |
| 16383 | -/C |  |  |  |  |  | ***** |  |  |  |
| 16391 | G/- |  |  |  |  | ***** |  |  |  |  |
| 16392 | G/- | ***** |  |  |  |  |  |  |  |  |
| 16394 | A/- |  |  |  |  |  | ***** |  |  |  |
| 16399 | C/- |  |  |  |  |  | ***** |  |  |  |
| 16457 | G/- |  |  |  |  |  |  |  | ***** |  |
| 16474 | G/- |  | ***** | ***** | ***** | ***** |  | ***** | ***** | ***** |
| 16478 | G/- |  |  |  |  |  | ***** |  |  |  |
| 16518 | G/C |  |  |  |  |  |  |  | ***** |  |
| 16519 | G/- | ***** |  |  |  |  |  |  |  |  |
| 16519 | T/- |  |  |  |  |  |  |  | ***** |  |
| 16549 | -/C |  |  |  | ***** |  |  | ***** |  |  |
| 16550 | -/C | ***** |  |  |  |  |  |  |  |  |
| 16759 | -/C | ***** |  |  |  |  |  |  |  |  |
| 16812 | C/- |  |  | ***** |  |  |  |  |  |  |

Supplementary Table 2. A breakdown of position and type of errors observed in the control sample HL60, both before and after homopolymeric correction. Positions in which errors were corrected in at least one of the two sequences are depicted in bold. Positions in which a new error previously not identified in the original sequences are marked with a ^x^ next to the position. Color coding is as follows: Green represents errors in a homopolymeric stretch (single nucleotide repeated 3 or more times. Example: CCCCC). Blue represents a disagreement between the base called from one strand to another. (Example A/T). Red represents errors in a dinucleotide repeat region (two nucleotides repeated at least twice. Example: ATAT). Yellow represents a single nucleotide outside of homopolymer regions present in one consensus sequence but not the other. Purple represents a single nucleotide repeat error (A single nucleotide repeated twice in one sequence but only once in the other. Example: GN/GG). Orange represents other errors, including a misalignment before or after a homopolymeric stretch, or a single varying nucleotide located within a homopolymeric stretch that was misaligned. Examples: AAATTT/AAAATT; AAAATAAAA/AAATAAAAA.

| Position | Variation | Native | Native Corrected | Enriched | Enriched Corrected |
| --- | --- | --- | --- | --- | --- |
| **71** | G/- | ***** |  | ***** |  |
| 150 | C/T |  |  | ***** | ***** |
| **291** | A/- | ***** |  | ***** |  |
| **308** | C/- | ***** |  | ***** |  |
| **309** | C/- | ***** |  | ***** | ***** |
| **315** | C/- | ***** |  | ***** |  |
| 316 | C/- | ***** | ***** | ***** | ***** |
| **362** | A/- | ***** |  | ***** |  |
| **425** | T/- | ***** |  |  |  |
| 439 | C/- | ***** | ***** | ***** | ***** |
| **460** | C/- | ***** | ***** | ***** |  |
| **499** | C/- | ***** |  | ***** |  |
| **516** | A/- | ***** |  |  |  |
| **573** | C/- | ***** |  | ***** |  |
| **574** | C/- |  |  | ***** |  |
| **748** | A/- | ***** |  | ***** | ***** |
| **807** | C/- | ***** |  |  |  |
| **960** | C/- | ***** |  | ***** |  |
| **961** | C/- | ***** | ***** | ***** |  |
| **966** | T/- | ***** |  |  |  |
| **998** | A/- | ***** |  | ***** | ***** |
| **999** | A/- | ***** |  |  |  |
| **1169** | A/- |  |  | ***** |  |
| **1515** | A/- |  |  | ***** |  |
| **1615** | T/- |  |  | ***** |  |
| **1806** | A/- | ***** |  | ***** |  |
| **1906** | C/- |  |  | ***** |  |
| **2080** | C/- |  |  | ***** |  |
| **2136** | A/- | ***** |  | ***** | ***** |
| **2157** | A/- | ***** |  | ***** | ***** |
| **2233** | A/- | ***** |  | ***** | ***** |
| **2463** | A/- | ***** |  | ***** | ***** |
| **2464** | A/- | ***** |  | ***** | ***** |
| **2492** | C/- |  |  | ***** |  |
| **2508** | A/- | ***** |  | ***** |  |
| **2806** | A/- | ***** |  |  |  |
| **2935** | G/- | ***** |  |  |  |
| 3107 | C/- | ***** | ***** | ***** | ***** |
| 3172 | C/- | ***** | ***** | ***** | ***** |
| **3385** | A/- | ***** |  |  |  |
| **3487** | C/- |  |  | ***** |  |
| **3569** | C/- | ***** | ***** | ***** |  |
| **3570** | C/- |  |  | ***** |  |
| **3589** | C/- | ***** | ***** | ***** |  |
| 3642^x^ | C/T |  | ***** |  |  |
| **3897** | C/- | ***** | ***** | ***** |  |
| **4061** | C/- | ***** | ***** | ***** |  |
| **4111** | C/- | ***** |  |  |  |
| **4141** | C/- | ***** |  | ***** |  |
| **4181** | A/- | ***** |  | ***** | ***** |
| 4253 | C/- | ***** | ***** | ***** | ***** |
| **4322** | C/- | ***** | ***** | ***** |  |
| **4479** | C/- | ***** | ***** | ***** |  |
| **4553** | T/- | ***** |  | ***** | ***** |
| **4610** | A/- | ***** |  | ***** | ***** |
| **4611** | A/- | ***** |  | ***** | ***** |
| **4799** | C/- | ***** | ***** | ***** |  |
| **4837** | C/- | ***** |  | ***** |  |
| **4883** | C/- | ***** |  |  |  |
| **5236** | C/T | ***** |  |  |  |
| **5251** | T/- | ***** |  | ***** |  |
| **5287** | A/- | ***** |  | ***** | ***** |
| **5406** | A/- | ***** | ***** | ***** |  |
| **5495** | T/- | ***** |  |  |  |
| 5751 | A/- | ***** |  | ***** | ***** |
| **5752** | A/- |  |  | ***** |  |
| **5758** | G/- | ***** |  |  |  |
| **5834** | A/- | ***** | ***** | ***** |  |
| **5899** | C/- | ***** |  | ***** |  |
| **6173** | C/- | ***** |  | ***** |  |
| **6189** | C/- |  |  | ***** |  |
| **6225** | T/- | ***** |  |  |  |
| **6384** | G/- | ***** |  |  |  |
| **6424** | C/- | ***** | ***** | ***** |  |
| **6450** | C/- |  |  | ***** |  |
| **6616** | T/- | ***** |  |  |  |
| **6697** | A/- | ***** |  | ***** | ***** |
| **6698** | A/- | ***** |  | ***** | ***** |
| **6946** | T/- | ***** |  |  |  |
| **7098** | C/- | ***** | ***** | ***** |  |
| **7401** | C/- |  |  | ***** |  |
| 7402 | C/- | ***** | ***** | ***** | ***** |
| **7398** | C/- |  |  | ***** |  |
| **7451** | A/- | ***** |  | ***** | ***** |
| **7452** | A/- | ***** |  |  |  |
| **7471** | C/- | ***** |  | ***** | ***** |
| **7513** | C/- | ***** |  | ***** |  |
| **7519** | A/- | ***** |  | ***** |  |
| **7532** | A/- | ***** |  | ***** |  |
| **7576** | A/- | ***** |  |  |  |
| **7629** | C/- | ***** | ***** | ***** |  |
| 7959 | C/- | ***** | ***** | ***** | ***** |
| **8032** | C/- | ***** |  | ***** |  |
| **8099** | A/- | ***** | ***** | ***** |  |
| **8156** | G/- | ***** | ***** | ***** |  |
| 8231 | C/- | ***** | ***** | ***** | ***** |
| **8237** | A/- | ***** |  | ***** |  |
| **8276** | C/- | ***** | ***** | ***** |  |
| **8285** | C/- | ***** | ***** | ***** |  |
| **8410** | C/- | ***** |  |  |  |
| **8447** | A/- | ***** |  |  |  |
| **8476** | C/- | ***** |  |  |  |
| **8495** | A/- | ***** |  |  |  |
| 8502 | A/- | ***** |  | ***** | ***** |
| **8562** | C/- | ***** |  |  |  |
| 8608 | C/- | ***** | ***** | ***** | ***** |
| **8609^x^** | C/- |  | ***** | ***** |  |
| **8760** | T/- | ***** |  |  |  |
| **8894** | A/- | ***** |  | ***** |  |
| 9437 | A/- | ***** | ***** | ***** | ***** |
| **9483** | T/- | ***** |  | ***** | ***** |
| **9484** | T/- | ***** |  | ***** | ***** |
| **9510** | T/- | ***** |  |  |  |
| **9537** | C/- | ***** |  |  |  |
| **9559** | C/- | ***** | ***** | ***** |  |
| **9585** | C/- |  |  | ***** |  |
| **9667** | A/- | ***** |  |  |  |
| **9800** | T/- | ***** |  | ***** | ***** |
| **10052** | A/- | ***** |  | ***** | ***** |
| **10053** | A/- | ***** |  |  |  |
| **10157** | A/- | ***** |  | ***** |  |
| **10196** | C/- | ***** |  | ***** |  |
| **10385** | A/- | ***** |  | ***** |  |
| 10476 | C/- | ***** | ***** | ***** | ***** |
| **10629** | C/- | ***** | ***** |  |  |
| **10818** | A/- | ***** |  | ***** | ***** |
| **10819** | A/- | ***** |  |  |  |
| **10872** | C/- | ***** | ***** |  |  |
| **10885** | T/- | ***** |  | ***** |  |
| **10920** | C/- |  |  | ***** |  |
| **10940** | C/- | ***** | ***** | ***** |  |
| **10952** | C/- | ***** | ***** | ***** |  |
| **10982** | C/- |  |  | ***** |  |
| **11037** | A/- | ***** |  | ***** | ***** |
| **11038** | A/- | ***** |  | ***** |  |
| **11231** | C/T |  |  | ***** |  |
| **11233** | T/- |  |  | ***** |  |
| 11234 | C/- |  | ***** | ***** |  |
| **11237** | C/T | ***** | ***** |  |  |
| **11431** | C/- | ***** |  |  |  |
| **11516** | C/- | ***** | ***** | ***** |  |
| **11677** | C/- | ***** | ***** | ***** |  |
| **11831** | T/- | ***** | ***** | ***** |  |
| **11872** | C/- | ***** |  | ***** |  |
| 12071 | Y/T | ***** | ***** | ***** | ***** |
| **12088** | C/- | ***** |  | ***** |  |
| 12104^x^ | C/- |  | ***** |  |  |
| 12197 | C/- | ***** | ***** |  |  |
| **12241** | C/- | ***** |  |  |  |
| **12379** | C/- |  |  | ***** |  |
| 12389 | C/- | ***** | ***** | ***** | ***** |
| **12390** | C/- | ***** |  | ***** |  |
| **12423** | A/- | ***** |  | ***** |  |
| **12424** | A/- | ***** |  | ***** |  |
| **12425** | A/- | ***** |  | ***** |  |
| **12436** | C/- | ***** |  | ***** |  |
| **13038** | C/- | ***** | ***** | ***** |  |
| **13132** | C/- |  |  | ***** |  |
| 13236 | A/- | ***** | ***** | ***** | ***** |
| **13237** | A/- | ***** |  | ***** | ***** |
| **13411** | A/- | ***** |  | ***** |  |
| **13453** | C/- | ***** |  |  |  |
| **13588** | C/- | ***** |  |  |  |
| **13650** | C/- | ***** |  |  |  |
| 13757 | C/- | ***** | ***** | ***** | ***** |
| **13758** | C/- |  |  | ***** |  |
| 13767 | C/- | ***** | ***** | ***** | ***** |
| 13786 | C/- | ***** | ***** | ***** | ***** |
| **13950** | C/- |  | ***** | ***** |  |
| **14079** | A/- | ***** | ***** | ***** |  |
| 14158 | C/T |  | ***** |  |  |
| 14159 | C/- | ***** | ***** | ***** | ***** |
| **14248** | C/- |  |  | ***** |  |
| **14267** | C/T | ***** |  |  |  |
| **14284^x^** | C/- |  | ***** | ***** |  |
| **14429** | C/- | ***** | ***** | ***** |  |
| 14493 | C/- | ***** | ***** | ***** | ***** |
| 14509 | A/- | ***** | ***** | ***** | ***** |
| **14510** | A/- | ***** |  | ***** | ***** |
| **14535** | C/- | ***** |  | ***** |  |
| **14592** | C/- | ***** |  |  |  |
| **14610** | T/- |  |  | ***** |  |
| **14715** | A/- | ***** |  | ***** |  |
| 14774 | C/- | ***** | ***** | ***** | ***** |
| **14812** | C/- | ***** |  |  |  |
| **15371** | C/- | ***** |  | ***** |  |
| **15540** | C/- |  |  | ***** |  |
| **15787** | T/- | ***** |  |  |  |
| **15944** | T/- | ***** |  |  |  |
| **15966** | A/- | ***** | ***** | ***** |  |
| **16036** | G/- | ***** | ***** | ***** |  |
| **16166** | A/- | ***** |  |  |  |
| **16188** | C/- | ***** | ***** | ***** |  |
| **16262** | C/- |  |  | ***** |  |
| **16366** | C/- | ***** |  | ***** |  |
| 16379 | C/- | ***** | ***** | ***** | ***** |
| **16380** | C/- |  |  | ***** |  |
| **16391** | G/- | ***** |  |  |  |
| **16474** | G/- | ***** |  | ***** |  |
| **16518** | G/- | ***** |  |  |  |
| **16549** | C/- |  |  | ***** |  |
